# From engagement to empowerment: integrating gamification and the Living Lab methodology into child-centered health innovation

**DOI:** 10.3389/fdgth.2025.1685146

**Published:** 2025-11-28

**Authors:** Abdolrasoul Habibipour

**Affiliations:** Information Systems, Luleå University of Technology, Luleå, Sweden

**Keywords:** gamification, living lab, healthcare, participatory design, child care

## Abstract

This article presents the design, development, and field testing of Save the World, a gamified healthcare application aimed at promoting health awareness and environmental literacy among children aged 8–10 years. Developed within the Horizon Europe SynAir-G project, the application combines game-based mechanics with the iterative Living Lab (LL) methodology to foster engagement, inclusivity, and real-world learning. The app was cocreated with children, parents, teachers, healthcare professionals, and developers through a multistakeholder, cocreative process involving workshops in Sweden and Denmark. Drawing on LL principles, such as stakeholder engagement, real-life experimentation, and continuous feedback, this research enhanced the contextual relevance and usability of game features while addressing ethical considerations and diverse user needs. The field-testing results show that the integration of the gamification and LL methodologies significantly improved user engagement, educational value, and technical performance. The study demonstrates how LL and gamification can reinforce one another in creating meaningful, child-centered digital innovations, aligning with broader European goals around sustainability, digital inclusion, and participatory design.

## 1 Introduction

The integration of digital technologies in healthcare and education has opened new avenues for enhancing user engagement and learning outcomes (1). One such innovative approach is the use of gamification, which involves incorporating game design elements into non-game contexts to motivate and engage users (2, 3). This article presents the development and field testing of “Save the World,” a gamified healthcare application designed to promote participation in healthcare innovation among children aged 8–10 years. The application was developed within the SynAir-G project, leveraging the Living Lab (LL) methodology to ensure user-centric design and continuous improvement based on stakeholder feedback. LL is an approach to managing open innovation processes in which quadruple helix actors engage in cocreating innovations in real-life, everyday use contexts (4, 5).

As sociotechnical systems, LLs are designed to integrate technological, social, and organizational elements, emphasizing the interaction between users, developers, and contextual conditions (6). This makes LLs particularly suited for child-centered health innovation, where social inclusivity, ethical sensitivity, and technological usability must be co-optimized.

The “Save the World” app combines gamification with healthcare to serve as both an educational platform and a health monitoring tool. By engaging children in real-life environmental scenarios, the app aims to raise awareness about environmental health and sustainability challenges. Through interactive mini-games, children navigate pollution crises in European cities, developing critical thinking and decision-making skills. The app's design emphasizes real-world contextual relevance, inclusivity, and usability, making it accessible to a diverse user base.

Researchers, such as Gatta et al. (7), have called for deeper investigations into how gamification can be effectively combined with participatory design approaches, such as LL, to support inclusive and meaningful innovation, particularly in educational and healthcare contexts. This research responds to such a call by offering empirical evidence on the mutual reinforcement between LL principles and game-based mechanics in the design of a child-centered health application.

The research aim of this study was to evaluate how gamification and the LL methodology can be combined to enhance engagement, learning, and user satisfaction in digital health innovation for children. Specifically, the study investigated the added value of each approach individually and in combination, using a staged development framework involving the following three levels: no gamification, gamification without LL, and gamification with LL. This design allowed for a nuanced analysis of how user engagement, cocreation, and educational outcomes evolve across different configurations. In doing so, the study contributes practical insights into how LL and gamification can mutually reinforce one another in the development of impactful digital tools in healthcare and education.

The LL methodology employed in this research is characterized by early and continuous stakeholder engagement, value cocreation, openness and transparency, iterative processes, real-life experimentation and evaluation, distributed decision-making, and social inclusivity (8). By involving multiple stakeholders, including children, parents, teachers, healthcare professionals, schools, developer companies, and universities, the project ensured that the app was designed to meet the needs and preferences of its users. The iterative cycles of design, testing, and refinement allowed for continuous feedback and improvements, enhancing the app's usability and effectiveness (9, 10).

This paper is structured as follows: the next section provides an overview of the research methodology, detailing the iterative LL approach and stakeholder engagement processes. The results section presents the findings from the field testing workshops, highlighting the app's usability, technical performance, inclusivity, and engagement outcomes. The discussion section explores the implications of the findings, addressing the benefits and challenges of integrating gamification within an LL framework and offering recommendations for future research and practice. Finally, the conclusion summarizes the key contributions of the study and outlines potential directions for further investigation.

## 2 Methods

**Figure 1:**
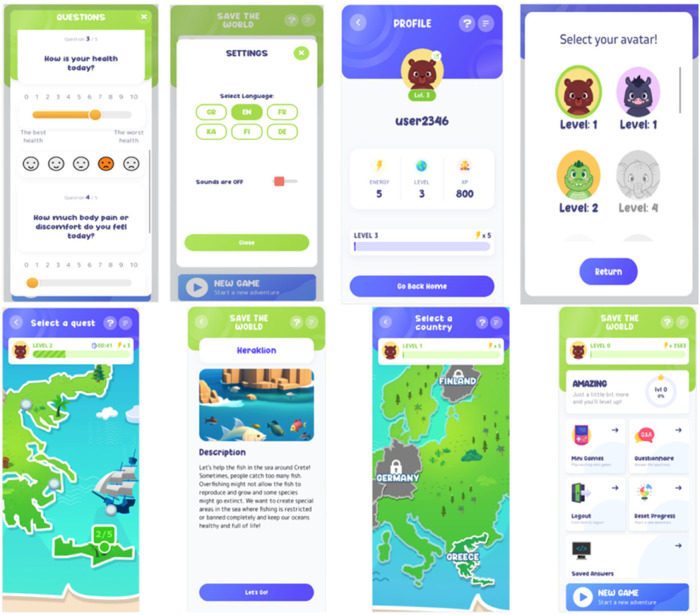
The Save the World game.

The development of the “Save the World” (see Figure 1) gamified healthcare application followed an iterative LL methodology, emphasizing cocreation in real-world settings and active engagement of multiple stakeholders. This approach ensured that the app was designed and refined based on direct user feedback and real-world testing, enhancing its relevance, usability, and effectiveness.

To evaluate the added value of the LL methodology and the role of gamification in a controlled manner, the project was designed around the following three distinct levels of development and testing: (1) a version of the app without any gamification elements, (2) a gamified version developed without applying LL principles, and (3) a gamified version cocreated and tested using a full LL methodology. This structure allowed for a comparative understanding of how gamification and LL each contribute to user engagement, usability, and educational value. At level one, participants interacted with a purely informational prototype to establish a baseline. Level two introduced game elements such as points, rewards, and interactive tasks without stakeholder co-creation. Level three, representing the final “Save the World” app, combined gamification with LL processes, including stakeholder cocreation, iterative testing, real-world contextualization, and inclusive design. This layered approach enabled an in-depth analysis of how LL principles enhance the meaningfulness and contextual relevance of gamified features, while also illustrating how gamification mechanics can serve as powerful tools to operationalize LL principles such as engagement, iteration, and inclusivity.

It is important to clarify that the three levels described above represent a developmental framework rather than a formal experimental design. Different prototypes were iteratively developed and evaluated during the design process. However, only the final version, which integrated both gamification and LL methodology, was tested by children during the field events in Sweden and Denmark. The earlier versions (non-gamified and gamified without LL input) were reviewed internally and refined in collaboration with educators and project stakeholders, but were not formally tested with the same children who participated in the final field-testing workshops. Therefore, the purpose of the three-level framework is to illustrate the evolution of the design process rather than to serve as a basis for direct comparison across participant groups. Future studies may build on this layered approach by implementing a formal experimental comparison.

The app's development was structured around iterative cycles of design, testing, and refinement. Each cycle began with the initial design, which was informed by preliminary research and input from key stakeholders, including children, parents, teachers, healthcare professionals, and developers. Early prototypes were created to visualize and test core functionalities and user interfaces. These prototypes were then tested in real-world settings through structured workshops and events, allowing users to interact with the app and provide feedback.

Stakeholder engagement was a critical component of the LL approach. Children aged 8–10 years, as primary users, were actively involved in testing the app and providing feedback on its usability, gameplay mechanics, and educational content. Parents and teachers contributed insights into the app's educational value, usability, and potential integration into learning environments. Healthcare professionals ensured that the app effectively promoted health awareness and monitoring. Technical teams and academic researchers collaborated to address technical challenges and incorporate user feedback into iterative improvements.

**Figure 2:**
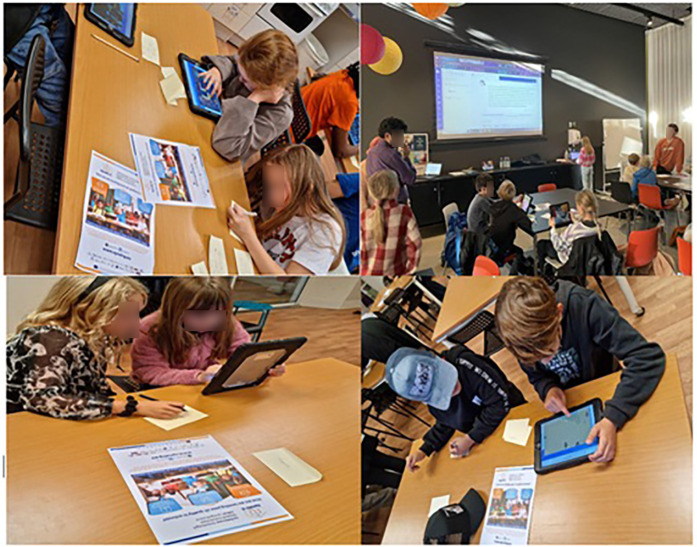
The two workshops.

Two field-testing sessions were conducted to gather user feedback and validate the app's design and functionality. The first event, held at the Luleå Science Centre (*Teknikens Hus*) during Researchers' Day (*Forskarfredag*) in Sweden, involved 28 children over a 3-h session. Participants tested the app and provided feedback through questionnaires, focusing on usability, technical performance, and educational content. The second event, organized at the “Next Generation Conference: Literacy of the Future” in Aarhus, Denmark, involved 24 children. Feedback from the Swedish event informed updates to the app, which were then validated during the Danish event. This brought the total number of child participants across both field-testing events to 52 (see Figure 2).

Participants were recruited via local schools and public science engagement events in Sweden and Denmark. Participation was voluntary, and informed consent was obtained from parents or guardians. The total sample included 52 children aged 8–10 years, with participation distributed across two countries. Participants were not randomized, and no formal stratification was conducted due to the exploratory nature of the study. The study did not include a power calculation, as its primary aim was user-centered design and usability evaluation rather than statistical inference. Therefore, the results should be interpreted as exploratory insights into user experience, rather than generalizable outcomes.

Feedback was collected through various methods, including structured questionnaires, observations, and informal interviews. Questionnaires gathered quantitative and qualitative data on user experiences, preferences, and suggestions. Researchers observed user interactions with the app to identify usability issues and areas for improvement. Informal interviews with children, parents, and teachers provided deeper insights into user experiences and expectations.

The posttest questionnaire was developed specifically for this study as part of the codesign process and reviewed by educators and healthcare professionals to ensure content relevance and age-appropriate language. It consisted of both closed and open-ended questions. The closed-ended items used a 1–10 Likert-type scale, where 1 represented the lowest or least favorable response and 10 represented the highest or most favorable. These questions assessed ease of use, enjoyment, engagement, clarity of instructions, audio experience, sense of accomplishment, technical performance, visual design, and overall impression of the app. Open-ended questions invited participants to name their favorite mini-game, describe any technical problems encountered, and suggest improvements. Responses were collected anonymously. The qualitative feedback was analyzed through informal collaborative review sessions among the research team, who discussed recurring themes and selected quotes that were representative of participant experiences. While the instrument was not statistically validated, it served the study's exploratory aims by offering insight into user perceptions and app usability in a real-world LL context.

Quantitative survey responses were analyzed using basic descriptive statistics, including means and frequency distributions. No inferential statistical tests were applied, as the primary goal was not to compare groups but to explore user perceptions of the final app prototype. Given the real-life, iterative nature of the LL methodology, this exploratory analysis was deemed sufficient to guide design refinement and assess overall user experience. Open-ended responses and observations were analyzed informally through collaborative team discussions during and after the workshops. Selected quotes included in the results section were chosen to reflect recurring patterns or particularly illustrative user experiences. While no formal coding or thematic analysis was performed, this approach was consistent with the formative and user-centered nature of the field testing.

Based on the collected feedback, the app underwent several rounds of updates and refinements. Technical enhancements addressed issues such as game freezes, navigation difficulties, and sound effects through backend updates and user interface improvements. Usability improvements included clearer instructions, simplified navigation, and enhanced accessibility features. Educational content and gameplay mechanics were adjusted to better align with user preferences and learning objectives.

The app's development followed three major design-test-refinement cycles over a period of approximately 6 months. After each cycle, feedback from children, teachers, and healthcare professionals was reviewed collaboratively by the research and development team. The first iteration focused on interface structure and core gameplay mechanics. Based on early feedback, the second iteration addressed issues such as confusing navigation, excessive sound effects, and insufficient instruction clarity. The final version integrated improvements to usability, accessibility, and educational alignment. The Swedish field-testing workshop took place in the early autumn of 2024, and the updated version was validated at the Danish event a few months later. These iterative refinements exemplify the LL principle of real-life experimentation and continuous improvement based on stakeholder input.

Ethical engagement practices were integral to the research methodology (11, 12). Transparent consent processes ensured that participants and their guardians were fully informed about the study's purpose and procedures. Rigorous data protection measures safeguarded participants' personal information and ensured compliance with ethical standards. The app's design prioritized inclusivity, considering cultural, linguistic, and gender diversity to create an accessible and engaging experience for all users.

By following this comprehensive and iterative LL methodology, the project successfully developed a gamified healthcare application that effectively engages young users in health monitoring and environmental awareness. The involvement of children as active contributors, supported by multistakeholder collaboration, highlights the potential of LL approaches to create impactful digital tools for healthcare and education.

## 3 Results

The development and field testing of the “Save the World” gamified healthcare application yielded significant insights into its usability, technical performance, inclusivity, and engagement outcomes. The iterative LL methodology facilitated continuous improvements based on user feedback, ensuring that the app met the needs and preferences of its young users. The quotes presented in the results were selected based on their relevance, clarity, and representativeness of common themes that emerged during the workshops.

*Usability and technical performance:* During the initial field-testing event in Sweden, several technical issues were identified, including game freezes, navigation difficulties, persistent sound effects, and unclear questionnaire requirements. These issues were addressed through updates to the app's backend, user interface, and help features. For instance, one participant noted, “The game freezes sometimes, making it hard to continue playing.” Another mentioned, “The navigation is confusing, and the sound effects are too loud.” These comments guided the technical enhancements that improved the overall user experience.

Subsequent testing in Denmark demonstrated enhanced user experiences, with fewer technical errors and improved navigation. Children praised the engaging gamified approach and educational content. One child remarked, “I love the mini-games! They are fun and teach me about the environment.” Parents and teachers also emphasized the importance of robust functionality and clear instructions. A teacher commented, “The app is a great tool for integrating health monitoring with environmental education, but it needs to be user-friendly.”

*Inclusivity and engagement:* The project prioritized inclusive design principles, ensuring that the app was accessible to a diverse user base. This included considerations for cultural, linguistic, and gender diversity. Feedback from the field-testing workshops highlighted the app's success in engaging children from different backgrounds. One parent noted, “It's wonderful to see an app that considers different languages and cultures. My child felt included and enjoyed playing the games.”

The testing events also highlighted important contextual factors. Some children faced challenges related to device access, especially those without personal smartphones or with parental restrictions. In addition, gender-based differences emerged in gameplay preferences; while boys tended to gravitate toward fast-paced, competitive games, girls often favored narrative or puzzle-based mini-games. These insights emphasize the need for inclusive design strategies that accommodate varied user contexts and preferences.

The app's gamified approach was particularly effective in maintaining user engagement. Children found the interactive quests and mini-games both enjoyable and educational. One participant stated, “The mini-games are so much fun! I learned a lot about pollution and how to solve environmental problems.” The sense of accomplishment provided by earning points and rewards was also well-received. A child mentioned, “I love earning points and seeing my progress. It makes me want to play more and learn more.”

### 3.1 Survey results

The posttest survey provided quantitative data on various aspects of the app. Participants rated their experience on a scale of 1–10 across multiple dimensions.

*Ease of use:* The main game received an average rating of 6.5, while the mini-games averaged 6.2. One child commented, “The mini-games are easy to understand and play.”

*Enjoyment:* The game's audio and sound effects were rated 6.4 on average. A participant noted, “The sounds are fun and make the game more exciting.”

*Engagement:* The activities within the game were rated 6.1 for engagement. One child mentioned, “The quests are interesting and keep me engaged.”

*Instructions:* The ease of following instructions received an average rating of 5.4. A participant suggested, “Better instructions would help understand the games better.”

*Technical issues:* The occurrence of technical issues was rated 4.8, indicating room for improvement. One child reported, “Sometimes the game freezes or doesn't respond.”

*Most enjoyable mini-games:* Participants identified their favorite mini-games, with “Memory Order,” “Deep Space Cleaning,” and “Parking Mayhem” being the most popular. One child stated, “Memory Order is my favorite because it's challenging and fun.”

*Suggestions for improvement:* Participants provided valuable feedback for further enhancements. Common suggestions included improving the controls, adding more levels, and providing better descriptions for the mini-games. One participant suggested, “More levels in the games, for example, parking mayhem, highway corners.” Another mentioned, “Better description for the mini-games. More levels in the games, for example, parking mayhem, highway corners.”

Table 1 shows the average user ratings across six key dimensions of app experience, providing a basic comparison between the participants in Sweden and Denmark.

**Table 1:** Summary of the children's feedback on different aspects of the game experience in Sweden and Denmark.

Dimension	Sweden (*n* = 28)	Denmark (*n* = 24)	Total (*N* = 52)
Ease of use (main game)	6.60	5.93	6.28
Ease of use (mini-games)	5.40	5.50	5.45
Enjoyment (sound effects)	5.53	6.00	5.76
Engagement	4.87	5.43	5.14
Clarity of instructions	6.13	4.64	5.41
Technical performance	4.87	4.71	4.79

*Ethical considerations:* Ethical engagement practices were integral to the research methodology. Transparent consent processes ensured that participants and their guardians were fully informed about the study's purpose and procedures. Rigorous data protection measures safeguarded the participants' personal information and ensured compliance with ethical standards. The app's design prioritized inclusivity, considering cultural, linguistic, and gender diversity to create an accessible and engaging experience for all users.

Overall, the iterative LL methodology proved effective in developing a user-centric healthcare application that successfully engaged young users in health monitoring and environmental awareness. The involvement of children as active contributors, supported by multistakeholder collaboration, highlights the potential of LL approaches to create impactful digital tools for healthcare and education.

## 4 Discussion

The integration of gamification within an LL framework, as demonstrated by the “Save the World” project, offers a promising approach to developing user-centric healthcare applications. This discussion explores the key aspects of integrating gamification in LL field tests, the benefits and challenges encountered, and the broader implications for future research and practice.

This research illustrates how LL methodologies and gamification can intersect to create more engaging, contextually grounded, and user-centered digital health solutions. Gamification mechanics such as feedback, rewards, and immersive challenges were instrumental in capturing and sustaining children's attention. Meanwhile, LL principles, including stakeholder cocreation, real-life testing, and iterative refinement, ensured that these mechanics were not only entertaining but also educationally and culturally relevant. The interaction between these approaches helped address key design tensions, such as balancing playfulness with meaningful content or ensuring inclusivity across diverse users and contexts.

*Integrating gamification into Living Lab field tests:* Gamification involves incorporating game design elements into non-game contexts to enhance user engagement and motivation (2). In the context of the “Save the World” project, gamification was used to create an educational and interactive platform for children to learn about environmental health and sustainability (13). The LL methodology facilitated the cocreation of the app by involving multiple stakeholders, including children, parents, teachers, healthcare professionals, and developers, in the design and testing process.

The iterative nature of the LL field tests allowed for continuous feedback and refinement of the app. Workshops and field-testing events provided opportunities for real-world testing, where children could interact with the app and provide valuable insights into its usability, technical performance, and educational content. This iterative process ensured that the app was tailored to the needs and preferences of its young users, enhancing its effectiveness and user satisfaction.

### 4.1 Benefits of gamification in the LL field tests

#### 4.1.1 Enhanced engagement

Gamification elements, such as points, rewards, and interactive quests, significantly increased user engagement. Children found the mini-games enjoyable and educational, which motivated them to participate actively in the app's activities. This aligns with existing research that highlights the positive impact of gamification on user engagement and motivation.

#### 4.1.2 Educational value

The gamified approach effectively conveyed complex environmental health concepts in an accessible and enjoyable manner. By navigating pollution crises and solving problems through mini-games, children developed critical thinking and decision-making skills. This demonstrates the potential of gamification to enhance learning outcomes in educational applications. While small incentives (e.g., symbolic gifts) were provided to children during the development process to encourage participation, the findings suggest that intrinsic motivators such as curiosity, learning, and environmental awareness were more influential drivers of engagement within the LL setting, especially when gamification was embedded in a real-life context (14). While the app was designed to promote awareness of environmental and health-related challenges, the study did not include a formal assessment of learning outcomes. Therefore, references to educational value are based on user perceptions, design intentions, and observed engagement, rather than measured knowledge gain.

#### 4.1.3 User-centric design

The LL methodology ensured that the app was designed with direct input from its primary users. This user-centric approach led to a more intuitive and user-friendly app, as evidenced by the positive feedback from children and their guardians. The involvement of diverse stakeholders also ensured that the app was inclusive and culturally relevant.

### 4.2 Challenges and considerations

#### 4.2.1 Technical issues

The initial field test workshops revealed several technical challenges, such as game freezes and navigation difficulties. Addressing these issues required iterative updates and refinements, highlighting the importance of robust technical development and testing processes in gamified applications.

#### 4.2.2 Balancing fun and education

While gamification can enhance engagement, it is crucial to balance the fun elements with educational content. Ensuring that the games are both enjoyable and informative requires careful design and continuous feedback from users. Some participants suggested adding more levels and providing clearer instructions to improve the educational value of the mini-games. Although the study refers to three levels of app development (no gamification, gamification without LL, and gamification with LL), these levels were intended as a conceptual framework to guide iterative design rather than a structure for experimental comparison. As such, no inferential statistical testing was applied. The field-testing workshops focused on the final version of the app, with usability and engagement insights derived from descriptive feedback and user responses. This approach aligns with the formative and user-centered nature of LL research, where the emphasis is placed on real-life relevance, participatory iteration, and contextual feedback rather than formal hypothesis testing.

#### 4.2.3 Ethical considerations

Ethical engagement practices are essential when involving children in research. Transparent consent processes, rigorous data protection measures, and inclusive design principles were integral to the “Save the World” project. Ensuring that children's data is securely handled and their participation is voluntary and informed is critical for responsible innovation.

#### 4.2.4 Sustaining user engagement

In real-life LL contexts, user engagement requires addressing both intrinsic and extrinsic motivational factors (15). While initial interest may be driven by novelty or small incentives, longer-term engagement depends on intrinsic motivators such as relevance, enjoyment, and learning value (10). Furthermore, as Mensink et al. (16) note, users often perceive the costs of participation as immediate, while the benefits remain uncertain or delayed, particularly in voluntary, non-commercial settings such as child-centered educational applications.

### 4.3 Living lab principles

The “Save the World” project exemplifies the application of key LL principles, which are essential for fostering effective and inclusive innovation.

#### 4.3.1 Early and continuous stakeholder engagement

The project involved stakeholders from the public sector, private sector, research institutions, and users/citizens throughout the development process. This ensured that diverse perspectives were considered, and the app was designed to meet the needs of all relevant stakeholders.

#### 4.3.2 Value cocreation

The collaborative design of the app emphasized value cocreation, delivering tangible and intangible benefits for all stakeholders. Children, parents, teachers, and healthcare professionals contributed to the app's development, ensuring that it was both educational and engaging.

#### 4.3.3 Openness and transparency

The project promoted openness and transparency by sharing knowledge and processes with all stakeholders. This built trust and encouraged collaboration, which was crucial for the app's success.

#### 4.3.4 Iterative processes

The iterative LL methodology embraced continuous cycles of experimentation, feedback, and refinement. This ensured that the app was inclusive and responsive to stakeholder needs, leading to a more user-friendly and effective product.

#### 4.3.5 Real-life experimentation and evaluation

The app was tested in real-life settings through workshops and field-testing events. This grounded the solutions in practical applicability and relevance, ensuring that the app was effective in real-world contexts.

#### 4.3.6 Distributed decision-making

The project shared power and decision-making among all stakeholders, promoting democratic governance and inclusivity. This approach ensured that all voices were heard and considered in the development process.

#### 4.3.7 Social inclusivity

The app's design actively identified and addressed the needs, motivations, and expectations of diverse stakeholders. This ensured that the solutions were equitable and inclusive, making the app accessible to children from different cultural and linguistic backgrounds.

### 4.4 Lessons learned

The “Save the World” project provided valuable lessons on the integration of gamification within an LL framework. One key lesson is the importance of early and continuous stakeholder engagement, which ensures that the app is designed to meet the needs of all relevant stakeholders. In addition, the iterative process of design, testing, and refinement proved crucial for addressing technical challenges and enhancing user experience. The project also highlighted the significance of ethical considerations, such as transparent consent processes and rigorous data protection measures, in fostering trust and ensuring responsible innovation. Finally, the success of the gamified approach in engaging and educating children underscores the potential of combining gamification with LL methodologies to create impactful digital tools for healthcare and education.

### 4.5 Implications for future research and practice

The successful integration of gamification within an LL framework in the “Save the World” project has several implications for future research and practice. First, the iterative LL methodology can be effectively applied to other healthcare and educational applications, fostering user-centric innovation. Second, the positive impact of gamification on engagement and learning outcomes suggests that similar approaches could be explored in various contexts, such as public health campaigns and environmental education programs.

Future research should continue to investigate the long-term effects of gamified applications on user behavior and learning outcomes. In addition, exploring the scalability of LL methodologies and gamification in different cultural and linguistic contexts can provide valuable insights into their broader applicability.

### 4.6 Synergistic power of Living Labs and gamification

The combination of the LL methodology and gamification fosters a powerful dynamic for digital health innovation. Gamification stimulates motivation through points, narratives, and interaction, while LLs ensure that this engagement is grounded in real-life contexts and shaped by users' needs. In this project, children, educators, and health experts codesigned and tested game features, contributing to better alignment between fun and function. Iterative testing (17, 18) helped refine elements such as challenge difficulty, learning outcomes, and navigation, leading to a product that was both educational and enjoyable. This synergy illustrates that gamification and LL are not separate layers but intertwined forces that, when integrated, can enhance both user experience and social relevance (2).

### 4.7 Study limitations

While this study offers valuable insights into the integration of gamification and the LL methodology in child-centered health innovation, several limitations should be noted. The number of participants, though sufficient for exploratory research in real-life settings, remains limited for drawing broader conclusions. The study was designed to test and refine a working prototype rather than compare outcomes across different versions of the application, and no control or comparison group was used. In addition, the duration of field testing was short and did not allow for observation of long-term learning outcomes or sustained engagement over time. Although the app was tested in two different national contexts, the cultural diversity within the participant group was not systematically recorded, which limits reflections on cross-cultural inclusivity. Finally, while the app's design process involved multiple stakeholder groups, the evaluation primarily captured children's perspectives and did not include systematic input from teachers or healthcare professionals after deployment. These limitations are inherent in many LL initiatives and point to important directions for future research. These limitations do not undermine the exploratory value of the study but rather highlight the contextual realities of conducting real-life testing in child-centered LL environments.

## 5 Conclusion

The “Save the World” project demonstrates the potential of integrating gamification within an LL framework to create engaging and educational healthcare applications for children. By involving multiple stakeholders throughout the development process, the project ensured that the app was user-centric, inclusive, and responsive to the needs of its young users. The iterative LL methodology facilitated continuous feedback and refinement, leading to significant improvements in usability, technical performance, and overall user experience. The positive reception from children, parents, and teachers underscores the effectiveness of gamification in enhancing engagement and learning outcomes, particularly in the context of environmental health and sustainability education.

The findings from this study highlight the importance of ethical engagement practices, inclusive design principles, and robust data protection measures in the development of digital tools for healthcare and education. The success of the “Save the World” app illustrates the value of early and continuous stakeholder engagement, value co-creation, and real-life experimentation in fostering user-centric innovation. Future research should explore the scalability of LL methodologies and gamification in diverse cultural and linguistic contexts, as well as the long-term impact of such applications on user behavior and learning outcomes. The insights gained from this project provide a valuable foundation for developing impactful digital solutions that empower young users to actively engage with health monitoring and environmental awareness. This approach also aligns with broader European Union priorities, including digital inclusion, environmental literacy, and child-centered innovation. In particular, it contributes to goals related to sustainability, health equity, and participatory design in nature-based solutions (NBS) and digital health strategies.

To conclude, this study offers empirical evidence on the mutual reinforcement between LL principles and game-based mechanics in the design of a child-centered health application. It contributes to the growing body of research at the intersection of gamification and LL methodologies by demonstrating how real-life experimentation, iterative cocreation, and stakeholder engagement can enhance the contextual, educational, and ethical relevance of gamified solutions. The findings also have practical implications for the development of inclusive, engaging, and policy-aligned digital tools, showing how LL frameworks can be leveraged to ground gamification in real user needs, particularly in educational and healthcare environments focused on sustainability and participation.

## Data Availability

The raw data supporting the conclusions of this article will be made available by the authors, without undue reservation.
